# Socioeconomic differences in dementia risk, lifestyle, and relevant determinants of behavior

**DOI:** 10.1177/13872877251414376

**Published:** 2026-02-09

**Authors:** Angelica D’Sa, Rik Crutzen, Markus Bödenler, Ana Diaz, Sten Hanke, Hannes Hilberger, Charlotta Thunborg, Francesca Mangialasche, Jeroen Bruinsma

**Affiliations:** 1 Department of Health Promotion of the Care and Public Health Research Institute, Maastricht University, Maastricht, The Netherlands; 2 SHE Research Centre, Department of Sport and Health Sciences, Technological University of the Shannon, Dublin Road, Athlone, Co. Westmeath, Ireland; 3 Institute of eHealth, University of Applied Science at FH Joanneum, Graz, Austria; 4 Alzheimer Europe, Senningerberg, Luxembourg; 5 GSRC, Division of Medical Physics and Biophysics, Medical University of Graz, Graz, Austria; 6 Division of Clinical Geriatrics, Center for Alzheimer Research, Department of Neurobiology, Care Sciences and Society, Karolinska Institutet, Stockholm, Sweden; 7 Theme Inflammation and Aging, Medical Unit Aging, Karolinska University Hospital, Stockholm, Sweden; 8 Department of Caring Sciences, Faculty of Health and Occupational Studies, University of Gävle, Gävle, Sweden; 9 FINGERS Brain Health Institute, Stockholm, Sweden

**Keywords:** Alzheimer’s disease, dementia, lifestyle, non-pharmacological interventions, population-based study, prevention, psychosocial interventions, public health, risk factors, socioeconomics

## Abstract

**Background:**

A healthy lifestyle supports cognitive aging while reducing dementia risk. Multidomain interventions promote healthy behavior, but are often unsuccessful in reaching those with a low socio-economic position (SEP), who face additional challenges with changing behavior.

**Objective:**

This cross-sectional study explores differences between SEP-groups in dementia risk, lifestyle, and the socio-cognitive determinants of behavior.

**Methods:**

3,341 Dutch adults (aged 40–79) were divided into low, medium, or high SEP groups. Using Chi-squared tests and ANOVA, SEP-related differences were explored for dementia risk, lifestyle behaviors, and health conditions. SEP-related differences in socio-cognitive determinants were examined using a modified version of Confidence Interval-Based Estimation of Relevance (CIBER).

**Results:**

Participants in the low SEP group had a significantly higher prevalence of all health conditions and engaged in more unhealthy behaviors, translating into a significantly higher dementia risk score. Many had misperceptions about the room for lifestyle improvement, but those in the low SEP group were slightly more aware of not adhering to lifestyle recommendations. Additionally, they perceived less self-confidence towards engaging in sports, considered healthy food as more expensive, perceived alcohol less pleasurable, experienced habits as less influential on alcohol intake, and had less confidence in their ability to quit smoking while pleasure and habits were strongly associated with smoking.

**Conclusions:**

Adults with a low SEP are at higher risk for dementia and have more potential for lifestyle-based risk reduction. Tailored, co-designed interventions that also consider the broader environment are needed to enhance perceived behavioral control, support behavior change, and reduce inequalities in dementia.

## Introduction

Globally, over 57 million individuals are living with dementia and this number is projected to almost triple by 2050, primarily due to a growing elderly population.^1,2^ This increase is placing strain on society and healthcare systems.^3^ Currently, there is no widely available curative treatment, and prevention strategies aimed to reduce dementia risk might offer a cost-effective strategy to mitigate the impact of dementia.^3,4^ Preventive lifestyle-based multidomain interventions have been developed, consistent with evidence that 45% of the global dementia cases are attributed to 14 modifiable factors, including various lifestyle behaviors and health conditions.^2^ Specifically, behaviors such as physical inactivity, poor diet, excessive alcohol use, smoking, low levels of cognitive and social activity, insufficient sleep, and poor adherence to cardiovascular treatments are contributing to dementia risk.^5–7^ Many of these behaviors elevate the risk for health conditions that further increase dementia risk, such as coronary heart disease, chronic kidney disease, diabetes, hypercholesterolemia, obesity, hypertension, depression, and hearing impairment.^5,8^

Social and economic factors—such as low education, limited income, harsh living environment, and challenging working conditions—are known to contribute to health inequalities, accounting for 30–55% of health outcomes.^9^ Individuals with a combination of these factors have a lower socio-economic position (SEP) and are more susceptible to engage in unhealthy lifestyle behaviors and develop health conditions,^10,11^ which leads to inequalities in dementia prevalence.^12–14^ Additionally, those with a low SEP more often have limited physical and psychological resources available to change lifestyle behaviors and manage health conditions.^11^ This stems from a complex interplay between individual and environmental factors that determine behavior and health.^15^ For instance, individuals with a low SEP often experience negative self-image and perceive limited control over initiating behavior change.^16^ These personal factors coincide with environmental challenges that complicate behavior change, such as financial stress or strenuous work conditions.^17^ Those with a low SEP are also known to benefit least from preventive endeavors,^11^ despite having the greatest potential for dementia risk reduction.^18^

Previously, we explored relevant socio-cognitive determinants of behavior in the context of dementia risk reduction. The findings indicate that many people have misperceptions and lack awareness of their room to improve lifestyle behavior to reduce dementia risk, while perceiving limited control over initiating change in behavior.^16^ In this study, we follow-up on these results and investigate differences between SEP-groups in dementia risk, lifestyle, and socio-cognitive factors that determine behavior. Currently, there is limited insight into socio-cognitive determinants of behavior change in the context of dementia risk. Examples of socio-cognitive determinants are attitudes, risk perceptions, and perceived control over changing specific behaviors. More understanding of SEP-related differences in determinants of behavior can support the design of appropriate interventions. Such interventions need to include behavior change methods that are able to target relevant socio-cognitive determinants to support individuals with low SEP with reducing dementia risk. Considering that lifestyle behavior is influenced by a complex interaction between individuals and their environment, the prevention of dementia requires a multi-level approach,^19,20^ encompassing both individual- and environmental-level strategies. In this study, we investigate dementia risk, behavior, socio-cognitive determinants of individuals, and explore how their socio-economic environment impacts this. Based on the findings, we provide future directions for individual-level interventions specifically, and where appropriate address implications for multi-level strategies to reduce dementia risk.

## Methods

### Study design

This cross-sectional study was conducted in the Netherlands. The study protocol was pre-registered (https://doi.org/10.17605/OSF.IO/GB4M5),^21^ and materials and data used in this study are openly available.^22^ To report the findings, we used the Strengthening the Reporting of Observational Studies in Epidemiology (STROBE) checklist.^23^

### Recruitment

In September 2022, participants were recruited through Flycatcher, a Dutch research agency with a large online panel and representation of the Dutch population. On voluntary basis, Dutch-speaking participants aged 40 to 79 years were recruited as dementia risk is associated with lifestyle behaviors during mid-life and late-life.^24,25^ A sample size estimation indicated that at least 4000 participants were required, details are described elsewhere.^16,21^

### Data collection

The screening questionnaire was based on the validated Lifestyle for Brain Health Index (LIBRA) dementia risk score.^26^ A self-developed follow-up questionnaire assessed socio-cognitive determinants of these behaviors. Both questionnaires were developed using an iterative process that is described elsewhere^16,21^ and involved the triangulation of literature, interviews,^27^ expert opinion, and refined based on a pre-test with individuals with a low SEP.

*Screening questionnaire*. The LIBRA index^26^ was used to identify respondents who could improve their lifestyle by changing behavior to reduce dementia risk. The screening questionnaire assessed various lifestyle behaviors and health conditions. Recommendations from Dutch lifestyle guidelines and earlier research were used in defining inclusion cut-offs to identify those with room for improvement in at least one lifestyle behavior (Table 1).

**Table 1.** Factors from lifestyle for brain health Index (LIBRA) and inclusion cut-offs.

Behavior	Measurement tool	Inclusion	Rationale
Physical Inactivity*	9–item Rapid Assessment of Physical Activity (RAPA)^28^	Less than 150 min of moderate-to-vigorous activity per week, spread over several days	Aligning with the Dutch lifestyle recommendations for physical activity^29^
Low adherence to Mediterranean diet*	14-item Mediterranean Diet Adherence Screener (MEDAS14)^30^	Score of ≤5 indicates low adherence to a Mediterranean Diet	Aligning with recommendations by the Dutch diet guidelines^31^
Overconsumption of alcohol*	Alcohol Use Disorders Identification Test (AUDIT)^32^	>7 units of alcohol per week on average	Aligning with the Dutch guidelines for alcohol intake^33^
Smoking*	Self-constructed item asking for the number of tobacco products consumed per day^16^	Current smokers	Smoking in mid-life or later-life is a known risk factor^34^
Low engagement in social and cognitive activities*	Self-constructed 12–item list that was based on previous research on dementia risk^35^	Below the median split of weekly activity in the sample	This cut-off has been used in previous research on dementia risk^36^
Health conditions	Self-reported medical history of coronary heart disease, renal dysfunction, diabetes, high cholesterol, hypertension, and depression	N/A	N/A
Obesity**	Body Mass Index based on self-reported height and weight	N/A	N/A

*These behaviors were used to select participants with room for lifestyle-related improvement in the context of dementia risk reduction. **Obesity was based on a BMI of 30 or higher.

*Follow-up questionnaire*. Participants with room for improvement in at least one behavior were invited for the follow-up questionnaire that explored determinants of behavior and change. This questionnaire assessed socio-cognitive determinants separately for all behaviors (i.e., physical activity, diet, alcohol use, smoking, and engagement in social and cognitive activities). Notably, determinants for alcohol use and smoking were only assessed if participants indicated that they engaged in these behaviors.

Items were based on determinants derived from multiple theoretical frameworks on behavior^37,38^ and inspired by definitions and measurement instructions^39^ as well as interviews with Dutch adults about behavior change to reduce dementia risk.^27^ Overall, for each behavior the following categories of determinants were assessed: (i) beliefs about engaging in sufficient levels of the behavior, (ii) intentions to change, (iii) attitudes, (iv) risk perceptions, (v) social and environmental influences, and (vi) perceived behavioral control.

### Analysis

*Socioeconomic position (SEP)*. Participants were divided into low, middle, and high SEP groups based on income and educational level. Income was based on 5 categories of the Dutch salary benchmarks, a Golden Standard from the Data & Insights Network and Statistics Netherlands, ensuring a representative sample.^40^ Specifically, income levels included (i) minimum (≤€14,100), (ii) below average (€14,101 to €36,500), (iii) approximately average (€36,501 to €43,500), (iv) 1–2 times the average (€43,501 to €73,000), and (v) 2 or more times the average (≥€73,001). Respondents who refrained from disclosing their income were excluded. Educational level was measured using 11 categories according to the Dutch Standard Classification of Education (SOI 21) of Statistics Netherlands.^41^

Scores on income and educational level were z-standardized and then averaged into a composite score. The score was categorized based on quartiles into low (≤25th percentile), medium (26th–74th percentiles), and high (≥75th percentile) SEP groups. This score was consistently used to explore SEP group differences in demographics and dementia risk—including health conditions and lifestyle behaviors—using Chi-squared tests and one-way ANOVA in SPSS. When these tests indicated an overall difference (i.e., p-value of less than .05), post-hoc comparisons were used to explore which SEP groups differed. For the Chi-squared test, we applied a Bonferroni correction to control for multiple comparisons (three tests: low versus medium, low versus high, and medium versus high), and considered p-values less than 0.0167 as significant. For ANOVA, we used Hochberg's GT2, if equal variances were assumed or Games-Howell if equal variances were not assumed. These post-hoc tests are most appropriate when sample sizes are unequal.^42^ To confirm age- and sex-adjusted differences in SEP and dementia risk, a post-hoc generalized linear model was performed using LIBRA scores as the outcome, SEP as factor, and age and sex as covariates, using robust standard errors.

*Socio-cognitive determinants of behavior.* In SPSS, the responses from the follow-up questionnaire were used to assess the proportion of participants who had misperceptions about the extent to which they engaged in specific lifestyle behaviors. Specifically, participants were asked about their thoughts on their current behavior; for example, “Do you think you perform enough physical activity every week?”. Possible answers were (i) yes, (ii) sometimes, and (iii) no. Similar questions were used for diet, alcohol consumption, and engagement in social and cognitive activities. Independent cross tables were constructed for each SEP group to assess the extent to which these perceptions aligned with participants’ actual engagement in behaviors, according to inclusion cut-offs. To illustrate, this provided insight into how many physically inactive participants perceived to perform enough physical activity.

The differences in SEP-related socio-cognitive determinants were examined using a modified version of Confidence Interval–Based Estimation of Relevance (CIBER) from the *behaviorchange* package in R (version 4.4.2).^43,44^ Since the original CIBER function does not allow for direct group comparisons, we replicated and modified it to consolidate the results of the three SEP groups into a single figure, facilitating direct visual comparison across SEP groups.

As in the original CIBER, diamond plots were used to visualize the mean scores on items measuring determinants (left pane) and their associations with the outcome behavior (right pane) for each group. In the left pane, diamonds represent the mean scores of determinant items, with widths corresponding to 99% confidence intervals. This visualization provides insight into the room for improvement at the determinant level. For example, increasing risk perception may be a relevant intervention target, but only if current perceptions are low or moderate and are associated with the outcome of interest. Therefore, the right pane displays zero-order correlations between each determinant and the outcome behavior, with diamonds again reflecting estimates and widths representing 95% confidence intervals. These correlations indicate whether determinants are meaningfully associated with the outcome, thereby informing their relevance for interventions. For instance, enhancing low or moderate risk perceptions would only be justified if these perceptions are also linked to the desired behavior (e.g., greater physical activity or reduced alcohol intake). Additionally, CIBER presents estimates of the 95% confidence intervals for explained variance (R^2^) of the determinants on outcome behavior using the R base lm function (y ∼ x1 + x2 + …). In this study, the explained variance was calculated separately for each SEP group.

*Secondary analysis.* To operationalize engagement in cognitive and social activities we initially calculated the number of weekly activities; however, this approach did not reveal any SEP-related differences, conflicting with our expectations and prior evidence.^18^ Internal consistency of the items was modest (McDonald's omega = 0.66), reflecting the broader challenge of measuring these activities adequately. Given the considerable diversity in how social and cognitive activities are operationalized in dementia risk research,^45^ we further explored SEP-related differences using an average score of the original Likert ratings. Although we regard this alternative as less precise, it reflects the many approaches commonly employed in studies,^45^ underscoring the need for more robust measurement approaches.

## Results

**Figure 1.**
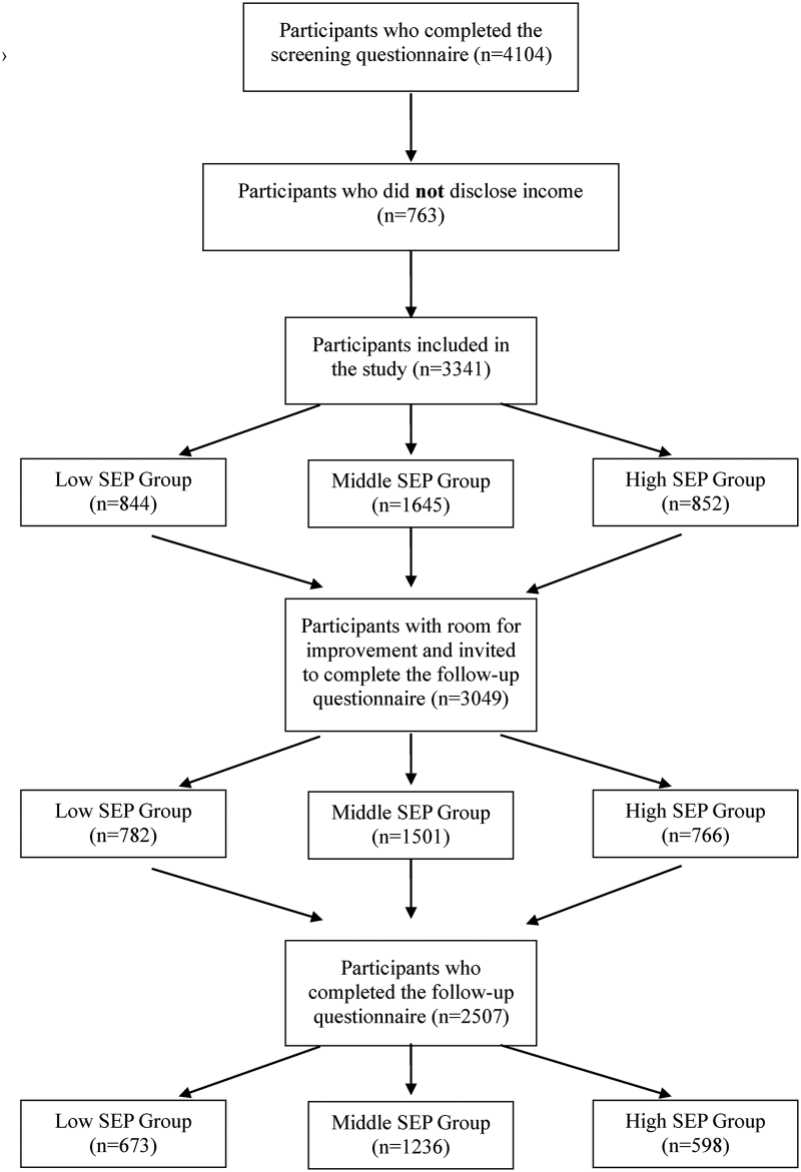
Flowchart of participant inclusion and follow-up by socioeconomic position. SEP: Socio-economic position.

Of the 4104 participants who completed the screening questionnaire, 18.6% (n = 763) did not disclose their income bracket and were not included in the CIBER analyses as SEP could not be determined (Figure 1). Compared to those who reported income, participants who did not disclose income were significantly older, more often female, and had a lower educational level. However, their dementia risk was lower (lower LIBRA total), they reported less alcohol consumption, and were more cognitively and socially active. No differences in the occurrence of health conditions were observed between participants who did and did not disclose income. These findings suggest that non-disclosure of income is not simply a reflection of lower SEP because in the low SEP group higher LIBRA scores and more frequent health conditions were observed (see Supplemental Table 1).

Of the 3341 included participants, 25.3% (n = 844) were classified as low SEP, 49.2% (n = 1645) as middle SEP, and 25.5% (n = 852) as high SEP (Figure 1). In the low SEP group, 92.7% (n = 782/844) had room to improve at least one behavior to reduce dementia risk. In the middle SEP group this was 91.2% (n = 1501/1645) and in the high SEP group 91.1% (n = 776/852). Participants with room for improvement were invited for the follow-up questionnaire with slightly higher response rates in the low SEP group (86.1%; n = 673/782), compared to the middle (82.3%; n = 1236/1501) and high SEP group (77.1%; n = 598/776).

### SEP-related differences in background, dementia risk, and lifestyle

Participants in the low SEP group were significantly older and more often female compared to those in other SEP groups (Table 2). Compared to the middle and high SEP groups, the low SEP group had a higher average LIBRA score, indicating a higher dementia risk. Post-hoc generalized linear models confirmed that SEP differences in LIBRA remained significant after adjustment for age and sex (see Supplemental Table 2).

**Table 2.** SEP differences in background characteristics and dementia risk factors.

	Low SEP (n = 844)	Middle SEP (n = 1645)	High SEP (n = 852)	p	Post-hoc
Age in years, mean (SD)	62.82 (9.75)	58.95 (10.30)	54.85 (10.55)	<0.001	All
Gender, female (%)	543 (64.3)	925 (56.2)	399 (46.8)	<0.001	All
LIBRA-factors,					
Physical inactivity					
n (%)^1^	462 (54.7)	881 (53.6)	429 (50.4)	0.163	/
mean RAPA (SD)	4.13 (1.01)	4.21 (0.93)	4.34 (0.78)	<0.001	LH; MH
Low Mediterranean diet					
n (%)^1^	551 (65.3)	885 (53.8)	376 (44.1)	<0.001	All
mean MEDAS (SD)	4.8 (1.97)	5.3 (2.06)	5.8 (1.98)	<0.001	All
Overconsumption of alcohol					
n (%)^1^	161 (19.1)	419 (25.5)	270 (31.7)	<0.001	All
mean units per week (SD)^2^	5.34 (8.50)	5.43 (7.20)	6.31 (8.26)	0.027	MH
Smoking					
n (%)^1^	135 (16.0)	194 (11.8)	54 (6.3)	<0.001	All
mean smokes per day (SD)^3^	13.9 (7.41)	11.9 (7.15)	8.8 (7.54)	<0.001	All
Low engagement in social and cognitive activities					
n (%)^1^	432 (51.2)	828 (50.3)	444 (52.1)	0.696	/
mean activities per week (SD)	18.5 (9.81)	18.3 (9.35)	18.4 (9.20)	0.935	/
post-hoc mean score (SD)*	3.23 (.87)	3.34 (.83)	3.43 (.78)	<0.001	All
LIBRA-score, mean (SD);	0.55 (3.05);	−0.31 (2.82);	−1.08 (2.66);	<0.001	All
min-max	−5.90 to 10.60	−5.90 to 9.10	-5.90 to 8.00		
Health conditions					
Coronary heart disease, n (%)	180 (21.3)	257 (15.6)	96 (11.3)	<0.001	All
Renal dysfunction, n (%)	27 (3.2)	27 (1.6)	14 (1.6)	0.022	LM
Diabetes, n (%)	135 (16.0)	158 (9.6)	53 (6.2)	<0.001	All
High blood cholesterol, n (%)	333 (39.5)	512 (31.1)	193 (22.7)	<0.001	All
Obesity, n (%)	261 (30.9)	355 (21.6)	127 (14.9)	<0.001	All
Hypertension, n (%)	359 (42.5)	581 (35.3)	217 (25.5)	<0.001	All
Depression, n (%)	196 (23.2)	311 (18.9)	106 (12.4)	<0.001	All

SEP: Socio-economic position. The LIBRA protective factors are inverted and represented as risk factors to enhance interpretability. ^1^Behaviors used to invite participants for the follow-up questionnaire. RAPA: Rapid Assessment of Physical Activity; MEDAS: Mediterranean Diet Adherence Screener. ^2^Mean units per week of participants who indicated to consume alcohol. ^3^Mean smokes per day of participants who indicated to smoke. For the post-hoc tests, LM indicates a significant difference between low and middle SES, LH between low and high SES, MH between middle and high SES, and All indicates that all SES groups differ significantly. *post-hoc conducted analysis.

All health conditions occurred more often in the low SEP group, where the adherence to a Mediterranean-like diet was lower and smoking occurred more frequently. No difference was observed in the rate of physical inactivity, but those in higher SEP were on average more physically active than those in low or middle SEP groups. Conversely, alcohol overconsumption was more frequent in the high SEP group compared to the low and middle SEP groups. Among those who consumed alcohol, the intake levels did not differ between the low with middle or high SEP groups.

### SEP-related differences in lifestyle perceptions

Overall, many misjudged their room for lifestyle improvement but participants in the low SEP group had slightly more accurate perceptions than those in the middle and high SEP groups. Those in low SEP more frequently responded “no”, and less frequently “yes”, to items asking if they engaged in sufficient physical activity, had a healthy diet, and were socially and cognitively active (Table 3). Conversely, an opposite trend was observed for alcohol overconsumption, where low SEP participants were less aware of their alcohol intake exceeding the recommendation of 7 glasses a week.

**Table 3.** Misperceptions of lifestyle behavior versus recommendations.

Do you think you do enough weekly physical activity?			
	Low SEP (n = 673)	Middle SEP (n = 1236)	High SEP (n = 598)
	Physical inactivity, n = 397	Physical inactivity, n = 724	Physical inactivity, n = 329
Yes, n (%)	154 (38.8)	282 (39.0)	128 (38.9)
Somewhat, n (%)	134 (33.8)	272 (37.6)	147 (44.7)
No, n (%)	109 (27.5)	170 (23.5)	54 (16.4)
Do you think you eat healthy?			
	Low SEP (n = 673)	Middle SEP (n = 1236)	High SEP (n = 598)
	Low Mediterranean diet, n = 472	Low Mediterranean diet, n = 729	Low Mediterranean diet, n = 295
Yes, n (%)	249 (52.8)	388 (53.2)	162 (54.9)
Somewhat, n (%)	191 (40.5)	314 (43.1)	120 (40.7)
No, n (%)	32 (6.8)	27 (3.7)	13 (4.4)
Do you think you overconsume alcohol?			
	Low SEP (n = 472)*	Middle SEP (n = 979)*	High SEP (n = 536)*
	Overconsumption of alcohol, n = 140	Overconsumption of alcohol, n = 352	Overconsumption of alcohol, n = 212
Yes, n (%)	19 (13.6)	33 (9.4)	30 (14.2)
Somewhat, n (%)	58 (41.4)	170 (48.3)	108 (50.9)
No, n (%)	63 (45.0)	149 (42.3)	74 (34.9)
Do you think that you are socially and actively engaged in life?			
	Low SEP (n = 673)	Middle SEP (n = 1236)	High SEP (n = 598)
	Low engagement in activities, n = 195	Low engagement in activities, n = 331	Low engagement in activities, n = 156
Yes, n (%)	77 (39.5)	171 (51.7)	94 (60.3)
Somewhat, n (%)	88 (45.1)	139 (42.0)	47 (30.1)
No, n (%)	30 (15.4)	21 (6.3)	15 (9.6)

SEP: Socio-economic position. Smoking was excluded as individuals who smoke are aware that they perform this behavior. *Completed only by participants who indicated they consume alcohol.

### SEP-related differences in socio-cognitive determinants

**Figure 2.**
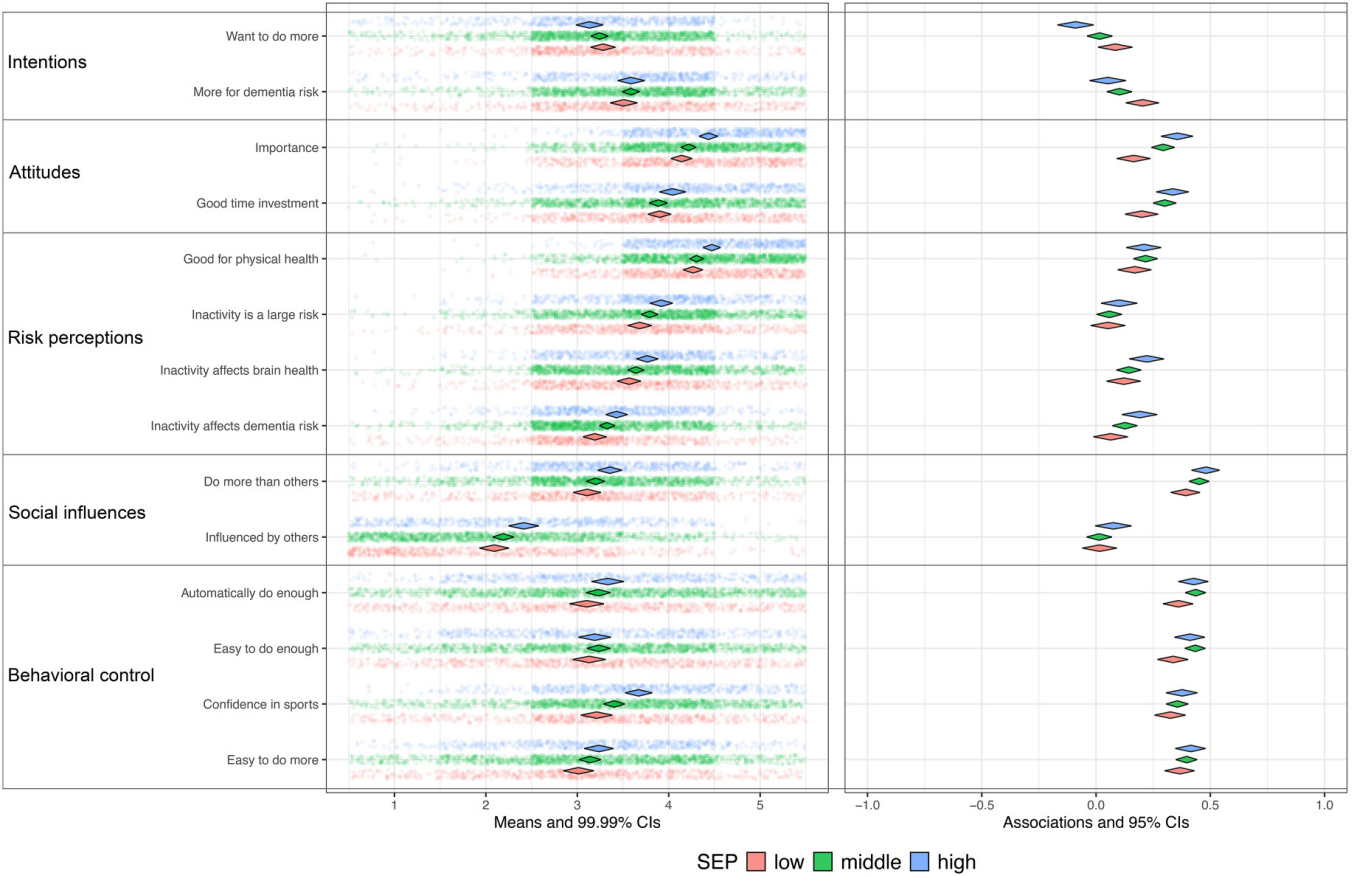
Physical activity and determinants for Low [R2: 0.16−0.27], Middle [R2: 0.23−0.31], High [R2: 0.26−0.38] SEP. SEP: Socio-economic position. Low (n = 673), Middle (n = 1236), High (n = 598). Left pane displays univariate distributions of determinant items and means with 99% CIs, showing room for improvement. Right pane displays zero-order correlations (r) between determinants and behavior with 95% CIs.

**Figure 3.**
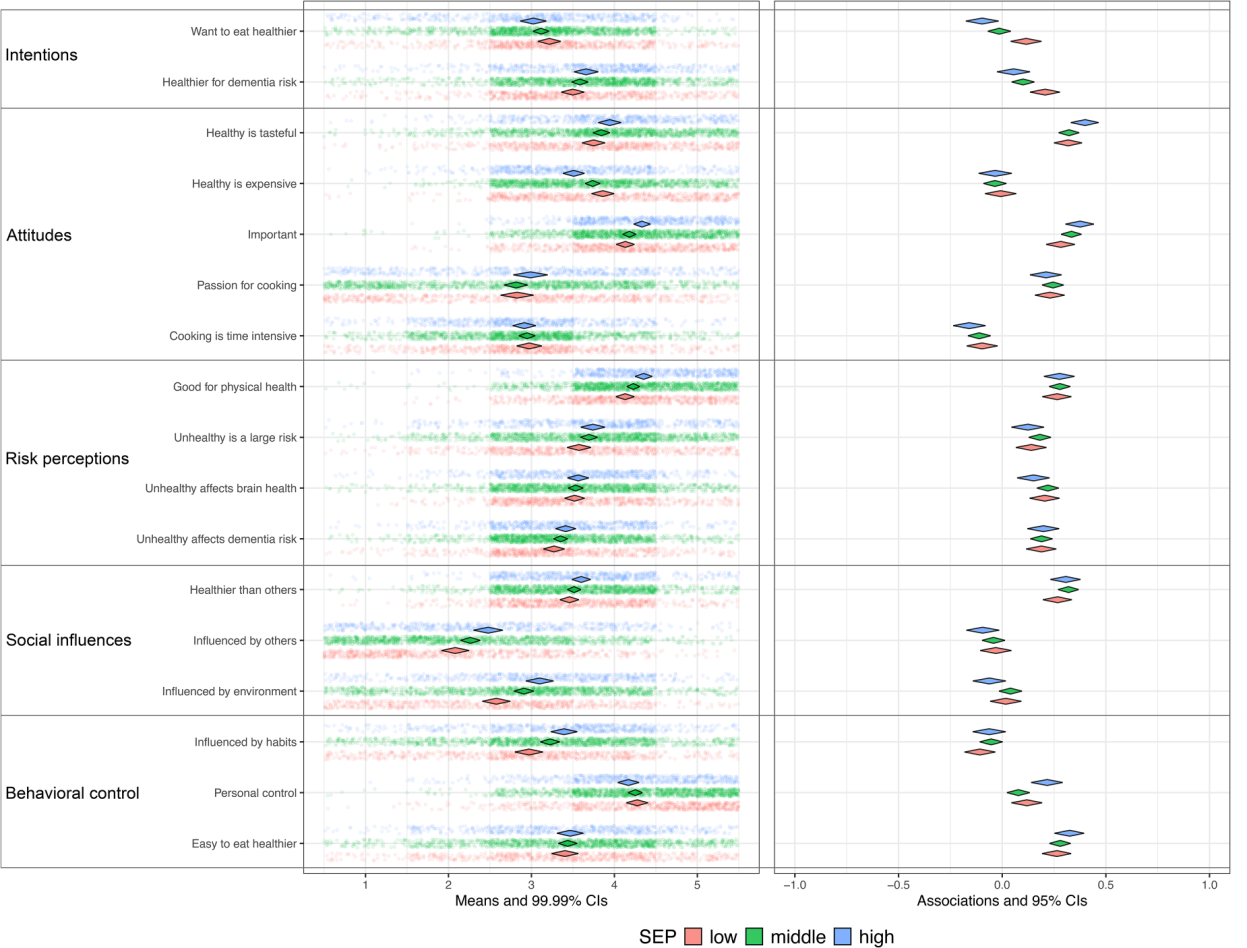
Mediterranean diet and determinants for Low [R2: 0.12−0.23], Middle [R2: 0.18−0.26], High [R2: 0.19−0.31] SEP. SEP: Socio-economic position. Low (n = 673), Middle (n = 1236), High (n = 598). Left pane displays univariate distributions of determinant items and means with 99% CIs, showing room for improvement. Right pane displays zero-order correlations (r) between determinants and behavior with 95% CIs.

**Figure 4.**
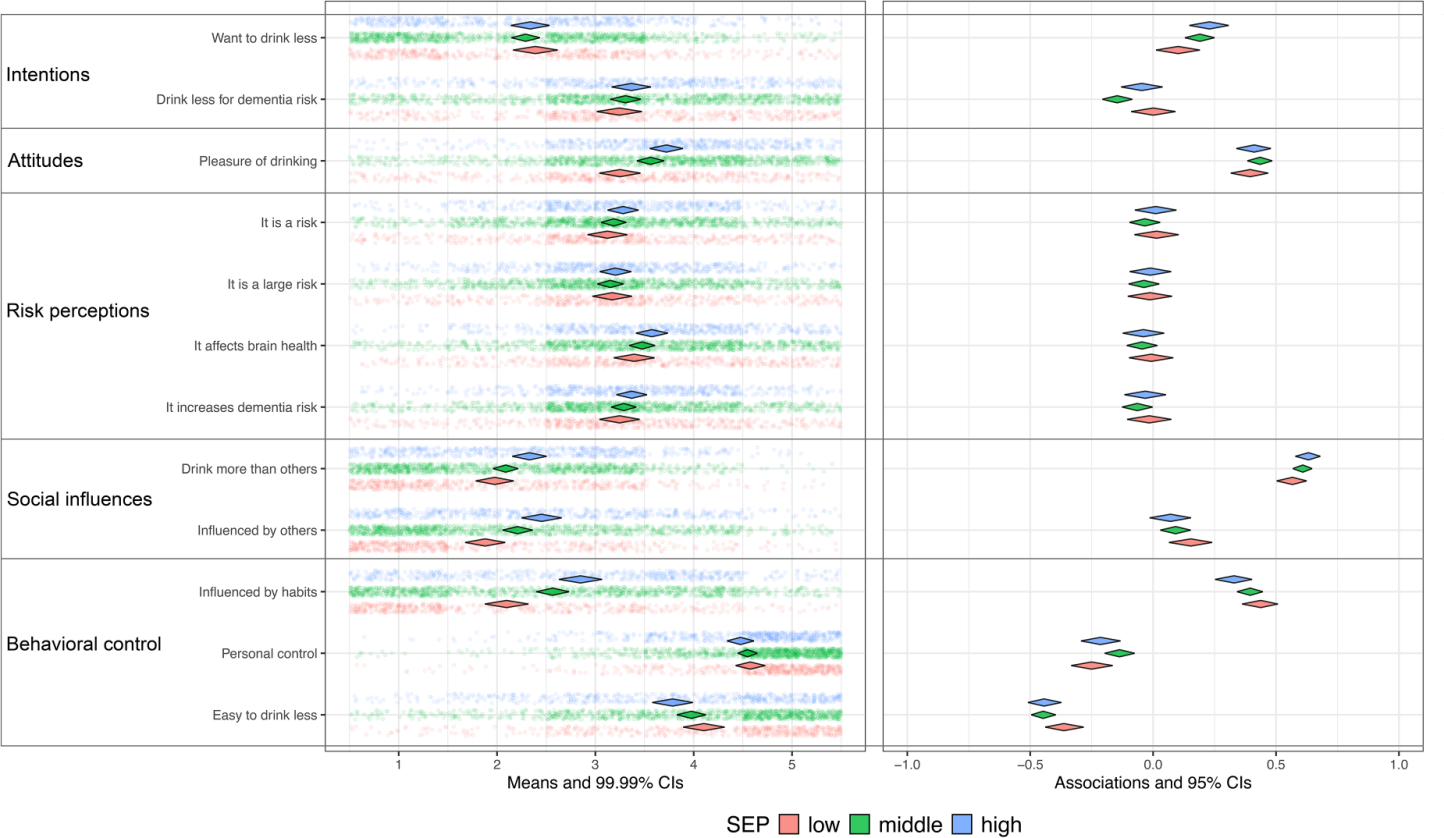
Consumption of alcohol and determinants for Low [R2: 0.33−0.47], Middle [R2: 0.39−0.48], High [R2: 0.37−0.5] SEP. SEP: Socio-economic position. Low (n = 472), Middle (n = 729), High (n = 295). Left pane displays univariate distributions of determinant items and means with 99% CIs, showing room for improvement. Right pane displays zero-order correlations (r) between determinants and behavior with 95% CIs.

**Figure 5.**
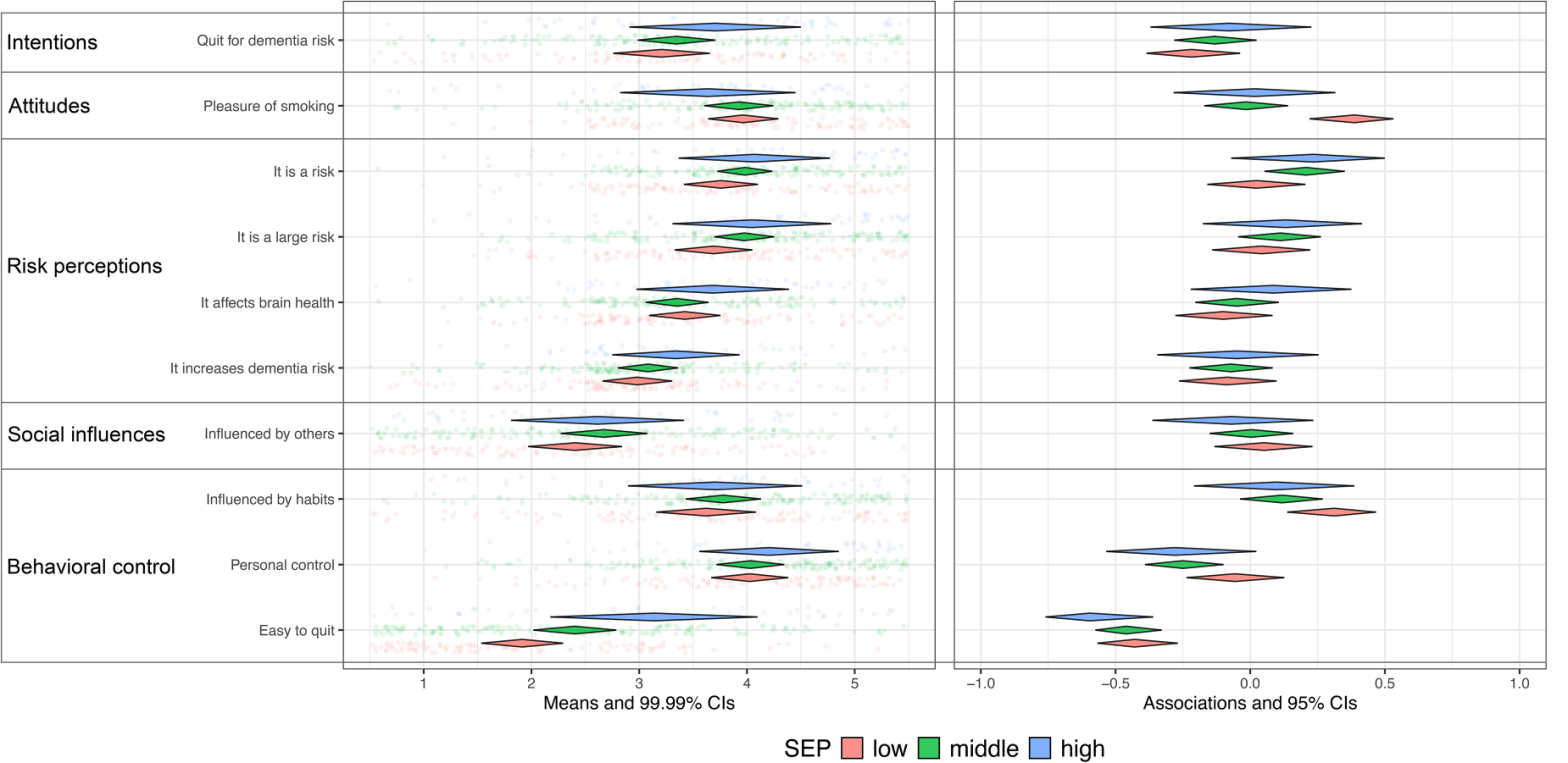
Smoking and determinants for Low [R2: 0.18−0.47], Middle [R2: 0.15−0.39], High [R2: 0.27−0.7] SEP. SEP: Socio-economic position. Low (n = 116), Middle (n = 159), High (n = 44). Left pane displays univariate distributions of determinant items and means with 99% CIs, showing room for improvement. Right pane displays zero-order correlations (r) between determinants and behavior with 95% CIs.

**Figure 6.**
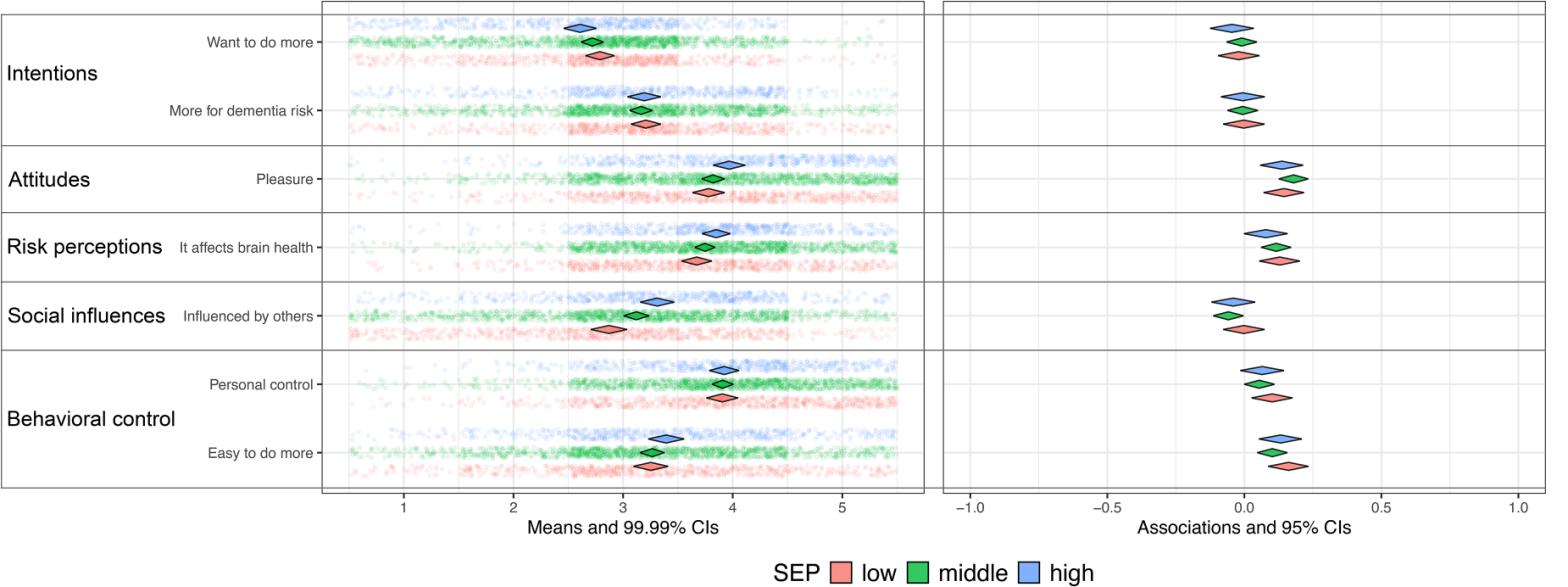
Social and cognitive activities and determinants for Low [R2: 0.02−0.08], Middle [R2: 0.03−0.07], High [R2: 0.01−0.06] SEP. SEP: Socio-economic position. Low (n = 673), Middle (n = 1236), High (n = 598). Left pane displays univariate distributions of determinant items and means with 99% CIs, showing room for improvement. Right pane displays zero-order correlations (r) between determinants and behavior with 95% CIs.

For each SEP group and per behavior a CIBER-based plot was created to allow for visual inspection of SEP related differences in the relevance of determinants, see Figures 2 to 6 (see Supplemental File 1).

The plots show that participants in the low SEP group felt less confident about engaging in sports compared to those in middle and high SEP groups, while in all groups this was strongly and positively associated with physical activity (Figure 2). This indicates its relevance with the greatest room for improvement in the low SEP group. Compared to the other groups, participants in low SEP perceived healthy eating as more expensive and considered their environment and habits as less influential on dietary patterns (Figure 3); however, these perceptions were only minimally associated with dietary behavior, which restricts the relevance. Regarding alcohol consumption, low SEP individuals experienced drinking alcohol as less pleasurable, and social influences and habitual patterns as less influential on their drinking behavior (Figure 4), compared to the other SEP groups. In all groups, these determinants were strongly associated with alcohol consumption, indicating high relevance. Participants in the low SEP group also had lower self-efficacy towards quitting smoking (Figure 5) and both habits and pleasure were stronger associated with smoking behavior, compared to other SEP groups. No relevant differences were observed for determinants related to the engagement in social and cognitive activities (Figure 6).

### Explained variance (R^2^)

For physical activity, dietary behavior, alcohol consumption, and smoking, the highest R^2^ was consistently found in the high SEP group, while—except for smoking—the lowest R^2^ was consistently observed in the low SEP group (Figures 2–6). This indicates that the socio-cognitive determinants included are generally less predictive of behavior in the low SEP group. For social and cognitive activities, the R^2^ was consistently low across all SEP groups (Figure 6).

## Discussion

### Key findings

In this study, we investigated SEP-related differences in dementia risk, lifestyle, and socio-cognitive determinants of behaviors in Dutch adults aged of 40 to 79 years. Our findings indicate that individuals with a low SEP have a higher risk for dementia and related health conditions and have more room to improve lifestyle behavior. Compared to those in middle or higher SEP, participants in the low SEP group had distinct patterns in socio-cognitive determinants, which have important implications for interventions.

### Interpretation

SEP profoundly shapes individuals’ opportunity for healthy aging through a complex interplay of social, economic, and environmental factors—not merely through deliberate choice.^46^ Individuals in a low SEP face additional barriers to change lifestyle behavior, including heightened (financial) stress, harsher living conditions, and limited access to resources that promote health and well-being.^10,17,47^ A lower SEP exacerbates challenges in prioritizing adherence to lifestyle recommendations and contributes to the development and progression of many health conditions.^11^ In turn and consistent with our findings, those in a low SEP have an increased risk for dementia.^18,19,25^

The relationship between SEP, lifestyle, and health conditions is complicated and likely to be multi-directional. For instance, certain health conditions may interfere with one's ability to work and are associated with the accumulation of care costs or sick leave, which can negatively impact income and consequently SEP, also known as downward social mobility.^48^ Additionally, health conditions may create barriers to maintaining or adopting healthy lifestyle behaviors, for example pain or fatigue. Our findings reveal that adults in the low SEP group more often have health conditions. In turn, these individuals may be confronted with limitations that hinder their ability to engage in certain lifestyle activities. This may explain why they were more often aware of not adhering to lifestyle recommendations.

Although unhealthy behaviors and health conditions were more prevalent among individuals in the low SEP group compared to the other groups, alcohol consumption was the exception to this pattern. Drinking more than the recommended 7 glasses a week was more prevalent in the high SEP group, however the averaged intake levels between the low and high SEP groups were not different. In Europe, the Netherlands is known for high alcohol intake, especially among those with a middle or higher educational level.^49^ This has been attributed to having more social opportunities to drink, as individuals with a high SEP have more financial means and opportunities for social gatherings.^50–52^ Additionally, the higher prevalence of health conditions in the low SEP group, could be indicative of the lower alcohol overconsumption rates as participants with health issues may deliberately refrain from drinking alcohol. Careful interpretation is needed, especially considering that individuals in a low SEP are more prone to alcohol-attributed health problems,^53,54^ and self-reported alcohol intake tends to be underestimated,^55,56^ tough it is unclear how SEP affects this. Given the widespread overconsumption of alcohol in the Dutch public,^49^ addressing alcohol intake across all SEP groups seems vital to promote healthy aging.

Overall, our findings highlight significant SEP-related differences in dementia risk, lifestyle, health conditions, and socio-cognitive determinants of behavior among middle-aged and older individuals in the Netherlands. As a high-income country with a robust healthcare and social welfare system, the Netherlands offers a context where disparities might be less pronounced compared to lower-income countries. In turn, SEP-related differences might be more pronounced in developing countries, which are also suggested to have the greatest potential for dementia risk reduction.^2^ Specifically, the highest increase in dementia cases is expected in low-income countries, while awareness of lifestyle prevention has been decreasing in these areas.^57^ Insights into country-specific SEP-related differences are crucial to develop strategies that are relevant within the context of these countries. Despite regional and national differences, dementia risk reduction strategies should be prioritized globally,^58^ as the vast majority of the public and healthcare professionals perceive dementia as a normal part of aging,^57^ which limits the implementation of preventive endeavors.

### Implications for interventions

Our findings underscore the importance of prioritizing the inclusion of low SEP populations in research and interventions for dementia risk reduction. A co-creation approach, involving stakeholders and individuals from low SEP communities, is needed to ensure dementia risk reduction endeavors become more relevant and effective for all segments of society. Individuals in the low SEP group had lower self-confidence towards engaging in sports activities and limited self-efficacy for quitting smoking. Therefore, individual-level interventions seem the most beneficial if perceived control can be enhanced through targeted behavior change methods,^59^ such graded goal setting and planning adequate coping responses to challenging situations. These methods should be included in carefully co-created interventions that resonate well with the perspectives and context of low SEP communities. Compared to those in middle or high SEP, adults in low SEP perceived healthy food as more expensive, which can hinder their ability to make healthier dietary choices. This perception likely also reflects the objective reality that healthier food options are often more costly, and these costs are relatively higher when considered against a lower income. Therefore, financial constraints may amplify the barriers to adopting healthier dietary patterns in low SEP groups. As shown in previous health promotion efforts specifically targeting low SEP communities, providing education on affordable food options and strategies can support healthier eating habits.^60^

Co-created individual-level interventions alone are probably not sufficient and broader environmental changes seem essential to tackle inequalities in dementia risk.^61^ For example, in the Netherlands, the cancellation of VAT increases on sports, coverage of smoking cessation programs through basic health insurance, and availability of food banks and social welfare benefits are steps towards reducing SEP-related barriers to healthier lifestyles. However, despite these actions, additional co-created efforts seem necessary to reduce inequalities in dementia and health. The recent proposal by the Dutch government to cut 300 million euros on prevention is a complication that contradict earlier commitments made in the Healthy and Active Living Agreement (*Gezond en Actief Leven Akkoord)* and the Integral Care Agreement (*Integraal Zorg Akkoord*). If not carefully addressed, such budget cuts can disproportionately impact the inclusion of vulnerable and underserved groups, potentially increasing disparities in health. In contrast, Singapore was recently recognized as a ‘blue zone’, with the highest health-adjusted life expectancy globally,^62^ as a result of specific preventative strategies such as promoting affordable public transport for all and subsidizing healthier food options. These examples from Singapore highlight that changing health disparities requires a multi-level approach supported by governmental commitments that account for the perspectives of stakeholders and the public.^20,61^

### Strengths and limitations

With a large sample, we could accurately estimate SEP-related differences in dementia risk, health conditions, lifestyle behavior, and socio-cognitive determinants of behavior. A considerable strength is that individuals with lower SEP were involved in the development of the questionnaires ensuring the appropriateness of items. Additionally, all materials and data are made openly accessible to support replication and future data synthesis.

In line with the descriptive nature of this study, we adjusted for multiple comparisons within tests but not across all outcomes (family-wise corrections). We recognize that this approach increases the chance of false-positive findings in terms of significance testing. However, the focus of our findings is on (accuracy of) estimation of parameters (i.e., means, correlations). Although our findings provide interesting implications, the cross-sectional nature of the study restricted our ability to investigate causal relationships between SEP, dementia risk, health conditions, lifestyle behavior, and determinants of behavior. However, such descriptive determinant studies are warranted.^63^ Additionally, it is a well-known challenge to include underrepresented groups in research,^64^ and it seems plausible that those with a low SEP experience more barriers to participate in online research panels. Participants with a low SEP demonstrated the highest response rates to the follow-up questionnaire, suggesting that once engaged they may be more likely to remain involved. Furthermore, the questionnaires were only available in Dutch, which limited the reach of the study and makes it challenging to draw conclusions for the entire Dutch population.

Further, 18.6% of our participants refrained from disclosing information about their income and were therefore excluded. These individuals had a lower educational level which may have influenced their inclusion and our conclusions, we recognize that individuals with a lower SEP may have more difficulty disclosing their income bracket. Additionally, educational level and income do not fully capture all elements of SEP, such as wealth, household income, financial scarcity, and occupational level.^65^ Notably, age is associated with lower educational level, and our low SEP group was significantly older, which may partially account for the higher prevalence of health conditions and unhealthier behavior observed in this group. Finally, the lower explained variance observed in most of our analyses for the low SEP group suggests that our measures may not fully capture all relevant determinants of behavior. This provides useful directions for future research to also explore the impact of interpersonal or environmental determinants—such as perceived safety, social cohesion, and neighborhood characteristics—that may better explain behavioral differences within lower SEP populations.

### Conclusion

Our findings highlight the need to address SEP-related disparities in dementia risk. Individuals with a lower SEP have a heightened risk for dementia development and the highest potential for lifestyle-related risk reduction. Carefully co-created interventions should focus on strengthening perceived behavioral control of initiating behavior change. Combining individual-level strategies with environmental improvements that accommodate healthier lifestyle behavior seems the most beneficial to reduce dementia risk across segments of the population.

## Supplemental Material

sj-docx-1-alz-10.1177_13872877251414376 - Supplemental material for Socioeconomic differences in dementia risk, lifestyle, and relevant determinants of behavior Supplemental material, sj-docx-1-alz-10.1177_13872877251414376 for Socioeconomic differences in dementia risk, lifestyle, and relevant determinants of behavior by Angelica D’Sa, Rik Crutzen, Markus Bödenler, Ana Diaz, Sten Hanke, Hannes Hilberger, Charlotta Thunborg, Francesca Mangialasche and Jeroen Bruinsma in Journal of Alzheimer's Disease

sj-docx-2-alz-10.1177_13872877251414376 - Supplemental material for Socioeconomic differences in dementia risk, lifestyle, and relevant determinants of behavior Supplemental material, sj-docx-2-alz-10.1177_13872877251414376 for Socioeconomic differences in dementia risk, lifestyle, and relevant determinants of behavior by Angelica D’Sa, Rik Crutzen, Markus Bödenler, Ana Diaz, Sten Hanke, Hannes Hilberger, Charlotta Thunborg, Francesca Mangialasche and Jeroen Bruinsma in Journal of Alzheimer's Disease
