# Supplementary material for: Socioeconomic differences in dementia risk, lifestyle, and relevant determinants of behavior
Source: J Alzheimers Dis. 2026 Feb 9;110(1):157–70. doi: 10.1177/13872877251414376 (PMC12960752; doi:10.1177/13872877251414376)
Supplement: sj-docx-1-alz-10.1177_13872877251414376 - Supplemental material for Socioeconomic differences in dementia risk, lifestyle, and relevant determinants of behavior [file sj-docx-1-alz-10.1177_13872877251414376.docx]

**Supplemental Material**

**Socioeconomic differences in dementia risk, lifestyle, and relevant determinants of behavior**

**Supplemental Table 1**. Comparing participants of whom data on income was not available (excluded) with those who provided their income bracket (included)

|  | Data on income | |  | |
| --- | --- | --- | --- | --- |
|  | Missing  (n =763) | Available  (n = 3,341) | | p |
| Age in years, mean (SD) | 60.20 (10.35) | 58.88 (10.61) | | 0.002 |
| Gender, female (%) | 527 (69.1) | 1,867 (55.9) | | <0.001 |
| Educational level, mean (SD) | 5.55 (2.77) | 5.94 (2.82) | | <0.001 |
| LIBRA-factors, |  |  | |  |
| Physical inactivity  n (%)^1^  mean RAPA (SD) | 400 (52.4)  4.22 (0.92) | 1,772 (53.0)  4.22 (0.92) | | 0.759  0.948 |
| Low Mediterranean diet  n (%)^1^  mean MEDAS (SD) | 425 (55.7)  5.29 (1.98) | 1,812 (54.2)  5.28 (2.05) | | 0.463  0.872 |
| Overconsumption of alcohol  n (%)^1^  mean units per week (SD)^2^ | 155 (20.3)  3.47 (5.83) | 850 (25.4)  4.48 (7.33) | | 0.003  <0.001 |
| Smoking  n (%)^1^  mean smokes per day (SD)^3^ | 73 (9.6)  12.88 (6.98) | 383 (11.5)  12.17 (7.46) | | 0.133  0.453 |
| Low social cognitive activity  n (%)^1^  mean activities per week (SD)  post-hoc mean score (SD)* | 353 (46.3)  18.90 (9.61)  3.38 (0.87) | 1,704 (51.0)  18.37 (9.43)  3.33 (0.83) | | 0.018  0.164  0.207 |
| LIBRA-score, mean (SD) | -0.609 (2.88) | -0.289 (2.90) | | 0.006 |
| Health conditions |  |  | |  |
| Coronary heart disease, n (%) | 119 (15.6) | 533 (16.0) | | 0.850 |
| Renal dysfunction, n (%) | 16 (2.1) | 68 (2.0) | | 0.914 |
| Diabetes, n (%) | 88 (11.5) | 346 (10.4) | | 0.340 |
| High blood cholesterol, n (%) | 219 (28.7) | 1,038 (31.1) | | 0.201 |
| Obesity, n (%) | 175 (23.0) | 743 (22.3) | | 0.676 |
| Hypertension, n (%) | 243 (31.8) | 1,157 (34.6) | | 0.144 |
| Depression, n (%) | 121 (15.9) | 613 (18.3) | | 0.105 |
| The LIBRA protective factors are inverted and represented as risk factors to enhance interpretability. ^1^Behaviors used to invite participants for the follow-up questionnaire. RAPA: Rapid Assessment of Physical Activity; MEDAS: Mediterranean Diet Adherence Screener. ^2^Mean units per week of participants who indicated to consume alcohol. ^3^Mean smokes per day of participants who indicated to smoke. For the post-hoc tests, LM indicates a significant difference between low and middle SES, LH between low and high SES, MH between middle and high SES, and All indicates that all SES groups differ significantly. *post-hoc conducted analysis. | | | | |

**Supplemental Table 2**. Post-hoc GLM to test for age- and sex-adjusted differences in SEP and dementia risk

|  | | |
| --- | --- | --- |
| **SEP group** | **Adjusted Mean** | **CI (lower ; upper)** |
| Low | 0.6248 | 0.4189 ; 0.8307 |
| Medium | -0.3086 | -0.4429 ; -0.1743 |
| High | -1.1580 | -1.3447 ; -0.9712 |

| **SEP group** | **Mean differences** | **CI** | **p** |
| --- | --- | --- | --- |
| Low versus Medium | 0.9334 | 0.6867 ; 1.1801 | <0.001 |
| Low versus High | 1.7828 | 1.4968 ; 2.0687 | <0.001 |
| Medium versus High | -0.8494 | 0.6202 ; 1.0785 | <0.001 |
