# Supplementary material for: Socioeconomic differences in dementia risk, lifestyle, and relevant determinants of behavior
Source: J Alzheimers Dis. 2026 Feb 9;110(1):157–70. doi: 10.1177/13872877251414376 (PMC12960752; doi:10.1177/13872877251414376)
Supplement: sj-docx-2-alz-10.1177_13872877251414376 - Supplemental material for Socioeconomic differences in dementia risk, lifestyle, and relevant determinants of behavior [file sj-docx-2-alz-10.1177_13872877251414376.docx]

Table 1 – Physical Activity: Means

| Determinant | Low SEP | | | Middle SEP | | | High SEP | | |
| --- | --- | --- | --- | --- | --- | --- | --- | --- | --- |
|  | M | Low | High | M | Low | High | M | Low | High |
| **PA_Change_T1** | 3.28 | 3.144 | 3.418 | 3.24 | 3.148 | 3.339 | 3.13 | 2.984 | 3.283 |
| **PA_Dementie_Risk_Change_T1** | 3.51 | 3.358 | 3.658 | 3.59 | 3.490 | 3.683 | 3.59 | 3.440 | 3.737 |
| **PA_Importance_T1** | 4.14 | 4.023 | 4.257 | 4.22 | 4.132 | 4.301 | 4.43 | 4.331 | 4.538 |
| **PA_Time_T1** | 3.90 | 3.771 | 4.027 | 3.89 | 3.785 | 3.988 | 4.04 | 3.896 | 4.188 |
| **PA_Importance_Health_T1** | 4.27 | 4.158 | 4.374 | 4.30 | 4.226 | 4.382 | 4.47 | 4.376 | 4.567 |
| **PA_Treath_T1** | 3.68 | 3.550 | 3.814 | 3.79 | 3.696 | 3.884 | 3.92 | 3.795 | 4.044 |
| **PA_Brains_T1** | 3.57 | 3.434 | 3.698 | 3.64 | 3.551 | 3.731 | 3.77 | 3.645 | 3.887 |
| **PA_Dementia_Risk_T1** | 3.19 | 3.062 | 3.321 | 3.33 | 3.240 | 3.412 | 3.43 | 3.309 | 3.550 |
| **PA_Social_Comparison_T1** | 3.11 | 2.954 | 3.260 | 3.20 | 3.098 | 3.303 | 3.36 | 3.222 | 3.490 |
| **PA_Social_T1** | 2.09 | 1.928 | 2.254 | 2.19 | 2.073 | 2.307 | 2.41 | 2.246 | 2.580 |
| **PA_Automatic_T1** | 3.10 | 2.913 | 3.292 | 3.23 | 3.095 | 3.364 | 3.33 | 3.155 | 3.511 |
| **PA_Difficult_T1_R** | 3.13 | 2.949 | 3.312 | 3.23 | 3.104 | 3.361 | 3.19 | 3.012 | 3.369 |
| **PA_Sport_Confidence_T1** | 3.21 | 3.038 | 3.384 | 3.40 | 3.284 | 3.521 | 3.67 | 3.521 | 3.820 |
| **PA_SelfEfficacy_T1** | 3.01 | 2.845 | 3.182 | 3.14 | 3.018 | 3.260 | 3.24 | 3.075 | 3.397 |

Table 2 – Physical Activity: Associations

| Determinant | Low SEP | | | Middle SEP | | | High SEP | | |
| --- | --- | --- | --- | --- | --- | --- | --- | --- | --- |
|  | ES | Low | High | ES | Low | High | ES | Low | High |
| **PA_Change_T1** | 0.08 | 0.009 | 0.159 | 0.02 | -0.040 | 0.071 | -0.09 | -0.169 | -0.010 |
| **PA_Dementie_Risk_Change_T1** | 0.20 | 0.130 | 0.275 | 0.10 | 0.047 | 0.157 | 0.05 | -0.029 | 0.131 |
| **PA_Importance_T1** | 0.16 | 0.090 | 0.238 | 0.29 | 0.242 | 0.344 | 0.36 | 0.284 | 0.424 |
| **PA_Time_T1** | 0.20 | 0.126 | 0.272 | 0.30 | 0.249 | 0.351 | 0.34 | 0.263 | 0.406 |
| **PA_Importance_Health_T1** | 0.17 | 0.094 | 0.242 | 0.22 | 0.163 | 0.269 | 0.21 | 0.132 | 0.285 |
| **PA_Treath_T1** | 0.05 | -0.024 | 0.128 | 0.06 | 0.002 | 0.113 | 0.10 | 0.022 | 0.180 |
| **PA_Brains_T1** | 0.12 | 0.046 | 0.196 | 0.14 | 0.089 | 0.198 | 0.22 | 0.145 | 0.298 |
| **PA_Dementia_Risk_T1** | 0.06 | -0.012 | 0.139 | 0.13 | 0.071 | 0.181 | 0.19 | 0.113 | 0.268 |
| **PA_Social_Comparison_T1** | 0.39 | 0.327 | 0.455 | 0.45 | 0.406 | 0.495 | 0.48 | 0.417 | 0.541 |
| **PA_Social_T1** | 0.02 | -0.060 | 0.091 | 0.01 | -0.042 | 0.070 | 0.08 | -0.004 | 0.155 |
| **PA_Automatic_T1** | 0.36 | 0.292 | 0.425 | 0.44 | 0.390 | 0.480 | 0.43 | 0.360 | 0.491 |
| **PA_Difficult_T1_R** | 0.34 | 0.269 | 0.403 | 0.43 | 0.388 | 0.478 | 0.41 | 0.343 | 0.476 |
| **PA_Sport_Confidence_T1** | 0.33 | 0.256 | 0.392 | 0.36 | 0.306 | 0.404 | 0.38 | 0.306 | 0.444 |
| **PA_SelfEfficacy_T1** | 0.37 | 0.299 | 0.431 | 0.40 | 0.349 | 0.443 | 0.42 | 0.346 | 0.479 |

Table 3 - MEDAS (Diet): Means

| Determinant | Low SEP | | | Middle SEP | | | High SEP | | |
| --- | --- | --- | --- | --- | --- | --- | --- | --- | --- |
|  | M | Low | High | M | Low | High | M | Low | High |
| **Diet_Change_T1** | 3.22 | 3.077 | 3.354 | 3.12 | 3.018 | 3.213 | 3.02 | 2.863 | 3.181 |
| **Diet_Dementie_Risk_Change_T1** | 3.50 | 3.359 | 3.637 | 3.58 | 3.485 | 3.683 | 3.66 | 3.512 | 3.806 |
| **Diet_Taste_T1** | 3.75 | 3.609 | 3.889 | 3.84 | 3.741 | 3.947 | 3.94 | 3.805 | 4.082 |
| **Diet_Costs_T1** | 3.86 | 3.724 | 3.997 | 3.74 | 3.647 | 3.829 | 3.51 | 3.380 | 3.640 |
| **Diet_Importance_T1** | 4.13 | 4.022 | 4.243 | 4.18 | 4.098 | 4.262 | 4.33 | 4.231 | 4.434 |
| **Diet_Fun_T1** | 2.83 | 2.627 | 3.025 | 2.81 | 2.670 | 2.954 | 2.99 | 2.781 | 3.195 |
| **Diet_Time_T1** | 2.97 | 2.821 | 3.126 | 2.95 | 2.848 | 3.046 | 2.91 | 2.773 | 3.053 |
| **Diet_Importance_Health_T1** | 4.13 | 4.018 | 4.241 | 4.23 | 4.149 | 4.308 | 4.35 | 4.249 | 4.457 |
| **Diet_Treath_T1** | 3.57 | 3.428 | 3.719 | 3.69 | 3.589 | 3.798 | 3.74 | 3.594 | 3.888 |
| **Diet_Brains_T1** | 3.52 | 3.399 | 3.642 | 3.53 | 3.440 | 3.625 | 3.56 | 3.434 | 3.693 |
| **Diet_Dementia_Risk_T1** | 3.27 | 3.144 | 3.400 | 3.35 | 3.266 | 3.438 | 3.41 | 3.290 | 3.536 |
| **Diet_Social_Comparison_T1** | 3.46 | 3.342 | 3.573 | 3.51 | 3.432 | 3.597 | 3.60 | 3.486 | 3.715 |
| **Diet_Social_T1** | 2.08 | 1.915 | 2.248 | 2.26 | 2.144 | 2.383 | 2.48 | 2.301 | 2.656 |
| **Diet_Environment_T1** | 2.58 | 2.408 | 2.745 | 2.91 | 2.788 | 3.030 | 3.10 | 2.931 | 3.266 |
| **Diet_Habits_T1** | 2.97 | 2.800 | 3.141 | 3.22 | 3.107 | 3.342 | 3.39 | 3.233 | 3.553 |
| **Diet_SelfControl_T1** | 4.27 | 4.144 | 4.406 | 4.25 | 4.165 | 4.338 | 4.17 | 4.039 | 4.299 |
| **Diet_SelfEfficacy_T1** | 3.40 | 3.243 | 3.566 | 3.44 | 3.322 | 3.553 | 3.47 | 3.306 | 3.627 |

Table 4 - MEDAS (Diet): Associations

| Determinant | Low SEP | | | Middle SEP | | | High SEP | | |
| --- | --- | --- | --- | --- | --- | --- | --- | --- | --- |
|  | ES | Low | High | ES | Low | High | ES | Low | High |
| **Diet_Change_T1** | 0.12 | 0.040 | 0.190 | -0.01 | -0.070 | 0.042 | -0.10 | -0.176 | -0.017 |
| **Diet_Dementie_Risk_Change_T1** | 0.21 | 0.134 | 0.279 | 0.10 | 0.045 | 0.156 | 0.05 | -0.025 | 0.134 |
| **Diet_Taste_T1** | 0.32 | 0.249 | 0.385 | 0.32 | 0.272 | 0.372 | 0.40 | 0.330 | 0.465 |
| **Diet_Costs_T1** | -0.01 | -0.083 | 0.068 | -0.04 | -0.091 | 0.020 | -0.03 | -0.114 | 0.046 |
| **Diet_Importance_T1** | 0.28 | 0.212 | 0.351 | 0.33 | 0.284 | 0.383 | 0.38 | 0.305 | 0.442 |
| **Diet_Fun_T1** | 0.23 | 0.158 | 0.301 | 0.25 | 0.192 | 0.297 | 0.21 | 0.134 | 0.287 |
| **Diet_Time_T1** | -0.10 | -0.171 | -0.022 | -0.11 | -0.166 | -0.056 | -0.16 | -0.237 | -0.080 |
| **Diet_Importance_Health_T1** | 0.26 | 0.193 | 0.334 | 0.28 | 0.226 | 0.329 | 0.28 | 0.201 | 0.349 |
| **Diet_Treath_T1** | 0.14 | 0.066 | 0.214 | 0.18 | 0.128 | 0.235 | 0.12 | 0.044 | 0.202 |
| **Diet_Brains_T1** | 0.21 | 0.132 | 0.277 | 0.22 | 0.167 | 0.273 | 0.15 | 0.072 | 0.229 |
| **Diet_Dementia_Risk_T1** | 0.19 | 0.115 | 0.261 | 0.19 | 0.135 | 0.243 | 0.20 | 0.121 | 0.275 |
| **Diet_Social_Comparison_T1** | 0.27 | 0.196 | 0.336 | 0.32 | 0.269 | 0.369 | 0.31 | 0.232 | 0.378 |
| **Diet_Social_T1** | -0.03 | -0.106 | 0.045 | -0.04 | -0.098 | 0.014 | -0.09 | -0.173 | -0.014 |
| **Diet_Environment_T1** | 0.02 | -0.058 | 0.093 | 0.04 | -0.016 | 0.095 | -0.06 | -0.142 | 0.018 |
| **Diet_Habits_T1** | -0.11 | -0.182 | -0.033 | -0.05 | -0.109 | 0.002 | -0.06 | -0.143 | 0.017 |
| **Diet_SelfControl_T1** | 0.12 | 0.044 | 0.193 | 0.08 | 0.022 | 0.133 | 0.22 | 0.139 | 0.292 |
| **Diet_SelfEfficacy_T1** | 0.26 | 0.192 | 0.333 | 0.28 | 0.228 | 0.330 | 0.33 | 0.252 | 0.395 |

Table 5 – Alcohol Consumption: Means

| Determinant | Low SEP | | | Middle SEP | | | High SEP | | |
| --- | --- | --- | --- | --- | --- | --- | --- | --- | --- |
|  | M | Low | High | M | Low | High | M | Low | High |
| **Alc_Change_T1** | 2.39 | 2.163 | 2.617 | 2.29 | 2.145 | 2.437 | 2.34 | 2.141 | 2.535 |
| **Alc_Dementia_Risk_Change_T1** | 3.24 | 3.013 | 3.474 | 3.31 | 3.150 | 3.463 | 3.37 | 3.165 | 3.567 |
| **Alc_Fun_T1** | 3.25 | 3.041 | 3.459 | 3.56 | 3.422 | 3.700 | 3.72 | 3.552 | 3.892 |
| **Alc_Health_T1** | 3.12 | 2.920 | 3.326 | 3.19 | 3.058 | 3.315 | 3.28 | 3.123 | 3.440 |
| **Alc_Treath_T1** | 3.17 | 2.970 | 3.373 | 3.15 | 3.018 | 3.287 | 3.21 | 3.044 | 3.367 |
| **Alc_Brains_T1** | 3.39 | 3.186 | 3.602 | 3.47 | 3.342 | 3.608 | 3.57 | 3.412 | 3.738 |
| **Alc_Dementia_Risk_T1** | 3.25 | 3.041 | 3.450 | 3.29 | 3.161 | 3.417 | 3.37 | 3.214 | 3.525 |
| **Alc_Social_Comparison_T1** | 1.98 | 1.782 | 2.171 | 2.09 | 1.959 | 2.217 | 2.33 | 2.155 | 2.506 |
| **Alc_Social_T1** | 1.88 | 1.675 | 2.083 | 2.21 | 2.055 | 2.364 | 2.45 | 2.248 | 2.658 |
| **Alc_Habit_T1** | 2.10 | 1.875 | 2.319 | 2.57 | 2.399 | 2.733 | 2.85 | 2.629 | 3.069 |
| **Alc_SelfControl_T1** | 4.58 | 4.425 | 4.727 | 4.55 | 4.447 | 4.648 | 4.48 | 4.338 | 4.613 |
| **Alc_SelfEfficacy_T1** | 4.10 | 3.893 | 4.315 | 3.98 | 3.828 | 4.125 | 3.79 | 3.578 | 3.993 |

Table 6 – Alcohol Consumption: Associations

| Determinant | Low SEP | | | Middle SEP | | | High SEP | | |
| --- | --- | --- | --- | --- | --- | --- | --- | --- | --- |
|  | ES | Low | High | ES | Low | High | ES | Low | High |
| **Alc_Change_T1** | 0.10 | 0.012 | 0.190 | 0.19 | 0.129 | 0.250 | 0.23 | 0.147 | 0.308 |
| **Alc_Dementia_Risk_Change_T1** | 0.00 | -0.089 | 0.091 | -0.15 | -0.207 | -0.084 | -0.05 | -0.130 | 0.039 |
| **Alc_Fun_T1** | 0.39 | 0.316 | 0.468 | 0.44 | 0.383 | 0.485 | 0.41 | 0.338 | 0.479 |
| **Alc_Health_T1** | 0.01 | -0.077 | 0.104 | -0.03 | -0.096 | 0.029 | 0.01 | -0.074 | 0.095 |
| **Alc_Treath_T1** | -0.01 | -0.103 | 0.077 | -0.04 | -0.100 | 0.025 | -0.01 | -0.096 | 0.074 |
| **Alc_Brains_T1** | -0.01 | -0.098 | 0.082 | -0.05 | -0.108 | 0.018 | -0.04 | -0.124 | 0.045 |
| **Alc_Dementia_Risk_T1** | -0.02 | -0.106 | 0.074 | -0.06 | -0.127 | -0.002 | -0.03 | -0.117 | 0.052 |
| **Alc_Social_Comparison_T1** | 0.57 | 0.502 | 0.625 | 0.61 | 0.568 | 0.647 | 0.63 | 0.578 | 0.680 |
| **Alc_Social_T1** | 0.15 | 0.064 | 0.240 | 0.09 | 0.029 | 0.153 | 0.07 | -0.014 | 0.154 |
| **Alc_Habit_T1** | 0.44 | 0.362 | 0.508 | 0.40 | 0.341 | 0.447 | 0.33 | 0.252 | 0.403 |
| **Alc_SelfControl_T1** | -0.25 | -0.334 | -0.164 | -0.14 | -0.198 | -0.075 | -0.21 | -0.294 | -0.132 |
| **Alc_SelfEfficacy_T1** | -0.36 | -0.439 | -0.282 | -0.45 | -0.496 | -0.396 | -0.44 | -0.509 | -0.373 |

Table 7 – Smoking: Means

| Determinant | Low SEP | | | Middle SEP | | | High SEP | | |
| --- | --- | --- | --- | --- | --- | --- | --- | --- | --- |
|  | M | Low | High | M | Low | High | M | Low | High |
| **Smoking_Dementia_Change_T1** | 3.21 | 2.758 | 3.655 | 3.35 | 2.984 | 3.707 | 3.70 | 2.909 | 4.500 |
| **Smoking_Pleasure_T1** | 3.97 | 3.638 | 4.293 | 3.92 | 3.605 | 4.244 | 3.64 | 2.822 | 4.450 |
| **Smoking_Harm_T1** | 3.76 | 3.414 | 4.103 | 3.98 | 3.726 | 4.237 | 4.07 | 3.366 | 4.771 |
| **Smoking_Treath_T1** | 3.69 | 3.329 | 4.050 | 3.97 | 3.697 | 4.253 | 4.05 | 3.308 | 4.783 |
| **Smoking_Health_T1** | 3.42 | 3.091 | 3.754 | 3.35 | 3.060 | 3.644 | 3.68 | 2.974 | 4.390 |
| **Smoking_Dementia_Risk_T1** | 2.98 | 2.659 | 3.306 | 3.08 | 2.801 | 3.362 | 3.34 | 2.750 | 3.931 |
| **Smoking_Social_T1** | 2.41 | 1.969 | 2.841 | 2.67 | 2.271 | 3.075 | 2.61 | 1.810 | 3.417 |
| **Smoking_Habits_T1** | 3.62 | 3.159 | 4.083 | 3.78 | 3.430 | 4.130 | 3.70 | 2.897 | 4.512 |
| **Smoking_SelfContol_T1** | 4.03 | 3.668 | 4.383 | 4.03 | 3.716 | 4.347 | 4.20 | 3.557 | 4.852 |
| **Smoking_SelfEfficacy_Quit_T1** | 1.91 | 1.533 | 2.295 | 2.40 | 2.017 | 2.788 | 3.14 | 2.175 | 4.098 |

Table 8 – Smoking: Associations

| Determinant | Low SEP | | | Middle SEP | | | High SEP | | |
| --- | --- | --- | --- | --- | --- | --- | --- | --- | --- |
|  | ES | Low | High | ES | Low | High | ES | Low | High |
| **Smoking_Dementia_Change_T1** | -0.22 | -0.385 | -0.037 | -0.13 | -0.282 | 0.024 | -0.08 | -0.370 | 0.227 |
| **Smoking_Pleasure_T1** | 0.39 | 0.220 | 0.532 | -0.02 | -0.170 | 0.141 | 0.02 | -0.284 | 0.316 |
| **Smoking_Harm_T1** | 0.02 | -0.159 | 0.205 | 0.21 | 0.053 | 0.351 | 0.23 | -0.071 | 0.500 |
| **Smoking_Treath_T1** | 0.04 | -0.141 | 0.223 | 0.11 | -0.045 | 0.263 | 0.13 | -0.176 | 0.415 |
| **Smoking_Health_T1** | -0.10 | -0.278 | 0.083 | -0.05 | -0.204 | 0.106 | 0.09 | -0.221 | 0.376 |
| **Smoking_Dementia_Risk_T1** | -0.09 | -0.264 | 0.098 | -0.07 | -0.226 | 0.084 | -0.05 | -0.345 | 0.254 |
| **Smoking_Social_T1** | 0.05 | -0.132 | 0.231 | 0.00 | -0.151 | 0.160 | -0.07 | -0.363 | 0.235 |
| **Smoking_Habits_T1** | 0.31 | 0.137 | 0.467 | 0.12 | -0.038 | 0.269 | 0.10 | -0.209 | 0.387 |
| **Smoking_SelfContol_T1** | -0.06 | -0.237 | 0.127 | -0.25 | -0.391 | -0.099 | -0.28 | -0.535 | 0.023 |
| **Smoking_SelfEfficacy_Quit_T1** | -0.43 | -0.568 | -0.268 | -0.46 | -0.575 | -0.329 | -0.60 | -0.760 | -0.360 |

Table 9 – Social Cognitive Activity: Means

| Determinant | Low SEP | | | Middle SEP | | | High SEP | | |
| --- | --- | --- | --- | --- | --- | --- | --- | --- | --- |
|  | M | Low | High | M | Low | High | M | Low | High |
| **Cog_Change_T1** | 2.79 | 2.657 | 2.921 | 2.72 | 2.616 | 2.822 | 2.61 | 2.457 | 2.757 |
| **Cog_Dementia_Risk_Change_T1** | 3.21 | 3.071 | 3.345 | 3.16 | 3.058 | 3.267 | 3.19 | 3.040 | 3.345 |
| **Cog_Fun_T1** | 3.78 | 3.633 | 3.927 | 3.82 | 3.715 | 3.927 | 3.97 | 3.821 | 4.115 |
| **Cog_Importance_Health_T1** | 3.67 | 3.532 | 3.811 | 3.75 | 3.653 | 3.840 | 3.85 | 3.720 | 3.979 |
| **Cog_Social_T1** | 2.87 | 2.704 | 3.037 | 3.12 | 3.004 | 3.237 | 3.31 | 3.156 | 3.466 |
| **Cog_SelfControl_T1** | 3.90 | 3.760 | 4.050 | 3.91 | 3.809 | 4.006 | 3.92 | 3.786 | 4.057 |
| **Cog_SelfEfficacy_T1** | 3.25 | 3.094 | 3.411 | 3.27 | 3.153 | 3.378 | 3.39 | 3.228 | 3.558 |

Table 10 – Social Cognitive Activity: Associations

| Determinant | Low SEP | | | Middle SEP | | | High SEP | | |
| --- | --- | --- | --- | --- | --- | --- | --- | --- | --- |
|  | ES | Low | High | ES | Low | High | ES | Low | High |
| **Cog_Change_T1** | -0.02 | -0.096 | 0.055 | -0.01 | -0.065 | 0.046 | -0.05 | -0.126 | 0.035 |
| **Cog_Dementia_Risk_Change_T1** | 0.00 | -0.077 | 0.074 | -0.01 | -0.062 | 0.050 | -0.01 | -0.086 | 0.075 |
| **Cog_Fun_T1** | 0.14 | 0.070 | 0.218 | 0.18 | 0.125 | 0.233 | 0.14 | 0.057 | 0.215 |
| **Cog_Importance_Health_T1** | 0.13 | 0.054 | 0.203 | 0.12 | 0.061 | 0.171 | 0.08 | -0.002 | 0.158 |
| **Cog_Social_T1** | 0.00 | -0.077 | 0.074 | -0.06 | -0.114 | -0.003 | -0.04 | -0.120 | 0.040 |
| **Cog_SelfControl_T1** | 0.10 | 0.026 | 0.176 | 0.05 | -0.001 | 0.110 | 0.07 | -0.015 | 0.145 |
| **Cog_SelfEfficacy_T1** | 0.16 | 0.087 | 0.234 | 0.10 | 0.047 | 0.157 | 0.13 | 0.053 | 0.210 |
